# Effect of duloxetine on changes in serum proinflammatory cytokine levels in patients with major depressive disorder

**DOI:** 10.1186/s12888-024-05910-0

**Published:** 2024-06-14

**Authors:** Wenfan Gao, Yejun Gao, Yayun Xu, Jun Liang, Yanhong Sun, Yuanyuan Zhang, Feng Shan, Jinfang Ge, Qingrong Xia

**Affiliations:** 1 Affiliated Psychological Hospital of Anhui Medical University, Hefei, China; 2 Department of Pharmacy, Hefei Fourth People’s Hospital, Hefei, China; 3 Psychopharmacology Research Laboratory, Anhui Mental Health Center, Hefei, China; 4 Anhui Clinical Research Center for Mental Disorders, Hefei, China; 5 Inflammation and Immune Mediated Diseases Laboratory of Anhui Province, Anhui Institute of Innovative Drugs, Anhui Medical University, Hefei, China; 6 The Key Laboratory of Anti-inflammatory and Immune Medicines, Ministry of Education, Hefei, China; 7 School of Pharmacy, Anhui Medical University, 81 Meishan Road, Hefei, 230000 PR China; 8 Department of Science and Education, Affiliated Psychological Hospital of Anhui Medical University, Hefei, China

**Keywords:** Pro-inflammatory cytokines, Serum, Duloxetine, Major depressive disorder, Patients

## Abstract

**Objective:**

Accumulating evidence supports the idea that inflammation may contribute to the pathophysiology of major depressive disorder (MDD). Duloxetine, a serotonin-norepinephrine reuptake inhibitor, exhibits anti-inflammatory effects both in vitro and in vivo. In this study, we investigated the impact of duloxetine on changes in serum proinflammatory cytokine levels among individuals diagnosed with MDD.

**Methods:**

A cohort of 23 drug-naïve individuals diagnosed with MDD and 23 healthy controls were included in this study. The severity of depressive symptoms was evaluated using the 24-item Hamilton Depression Scale (HAMD-24). A panel of 7 proinflammatory cytokines, including interleukin-1β (IL-1β), IL-2, IL-6, IL-8, IL-12, tumor necrosis factor-α (TNF-α), and interferon-γ (IFN-γ), were quantified using multiplex Luminex assays. The levels of serum cytokines in healthy controls and patients with MDD were compared at baseline. All patients received duloxetine at a dosage range of 40–60 mg/day for a duration of 4 weeks. The HAMD-24 scores and serum cytokine levels were compared before and after duloxetine treatment.

**Results:**

Compared with healthy controls, patients with MDD had significantly greater levels of IL-2, IL-6, IL-8, IL-12, TNF-α, and IFN-γ (*P* < 0.05). Moreover, there was a significant decrease in HAMD-24 scores observed pre- and post-treatment (*t* = 13.161, *P* < 0.001). Furthermore, after 4 weeks of treatment, the serum levels of IL-8 (*t* = 3.605, *P* = 0.002), IL-12 (*t* = 2.559, *P* = 0.018), and IFN-γ (*t* = 3.567, *P* = 0.002) decreased significantly. However, there were no significant differences in other cytokines, including IL-1β, IL-2, IL-6, and TNF-α, before and after treatment (*P* > 0.05).

**Conclusions:**

These findings present compelling evidence, potentially for the first time, indicating that duloxetine treatment may effectively reduce the serum concentrations of IL-8, IL-12, and IFN-γ in individuals diagnosed with MDD. However, the precise mechanisms underlying this effect remain unclear and warrant further investigation.

## Introduction

Depression, ranked by the World Health Organization as the primary cause of global disability, is widely acknowledged as a prevalent mental disorder [1]. The most recent comprehensive investigation of mental disorders in China revealed a lifetime prevalence rate of 6.9% for depression [2]. Extensive research has shown that depression frequently coincides with the activation of the inflammatory system, and the excessive expression of proinflammatory factors is believed to contribute to the development of depression [3, 4]. A meta-analysis demonstrated that interleukin-6 (IL-6), C-reactive protein (CRP), and tumor necrosis factor-α (TNF-α) are the inflammatory factors most closely associated with depression [5, 6]. Moreover, it has been reported that elevated plasma levels of inflammatory markers (IL-1 and IL-6) in older individuals are predictive of the development of depressive symptoms over a 6-year period [7]. In addition, the efficacy of celecoxib, a nonsteroidal anti-inflammatory drug (NSAID), in ameliorating postpartum depressive symptoms has been established [8]. Therefore, the potential correlation between inflammation and depression presents an avenue for addressing the latter through the implementation of anti-inflammatory strategies that target the immune system.

Recent research has provided evidence that both subchronic and acute administration of commonly prescribed antidepressants exhibit anti-inflammatory effects. Specifically, studies have shown that selective serotonin reuptake inhibitors (SSRIs), such as paroxetine and sertraline, can inhibit the increase in interferon-γ (IFN-γ)-induced TNF-α and nitric oxide (NO) in a murine microglial cell line [9]. Another investigation suggested that the anti-inflammatory effects of antidepressants may be attributed to their ability to modulate immune balance by suppressing the IFN-γ/IL-1 ratio [10]. Serotonin and norepinephrine reuptake inhibitors (SNRIs) can prevent depression-like behavior and changes in serum pro-inflammatory cytokines induced by lipopolysaccharide (LPS) in mice [11]. Furthermore, a clinical study demonstrated that patients with major depressive disorder (MDD) who received antidepressant treatment in clinical settings experienced notable improvement of symptoms and notable reductions in TNF-α levels [12]. Consequently, it is plausible that the anti-inflammatory properties of antidepressants may play a role in their therapeutic efficacy for MDD patients.

Duloxetine, an approved selective serotonin-norepinephrine reuptake inhibitor (SNRI), has been authorized for the treatment of depression in the United States and Europe since 2004 [13]. Currently, there is a paucity of research examining the longitudinal impact of duloxetine on a range of proinflammatory cytokines in individuals with MDD. Therefore, in this study, the effects of duloxetine on alterations in serum proinflammatory cytokines in patients with MDD were investigated. The levels of seven proinflammatory cytokines, namely, IL-1β, IL-2, IL-6, IL-8, IL-12, TNF-α, and IFN-γ, were measured. The severity of depressive symptoms was assessed using the 24-item Hamilton Depression Scale (HAMD-24). All patients received duloxetine treatment at a dosage range of 40–60 mg/day for four weeks. Subsequently, the serum cytokine levels before and after treatment were compared.

## Materials and methods

### Study design and participants

This study was carried out at Anhui Mental Health Center from August 2020 to June 2022. Twenty-three drug-naïve patients with MDD were diagnosed by trained psychiatrists based on the Diagnostic and Statistical Manual for Psychiatric Disorders-Fifth Version (DSM-V). The inclusion and exclusion criteria for patients are presented in Table 1. All participants were Han Chinese individuals residing in Anhui Province. The severity of depressive symptoms in all subjects was assessed using the Chinese version of the HAMD-24, which has demonstrated good reliability with a Cronbach’s alpha value of 0.714 [14]. The diagnostic criteria for MDD were a HAMD-24 score exceeding 20 points. A control group consisting of twenty-three individuals without MDD was included in the study. The research protocol was approved by the ethics committee of the Anhui Mental Health Center, with registration number HFSY-IRB-PJ-XQR-2,020,001, and adhered to the principles outlined in the Declaration of Helsinki. Informed consent was obtained from all participants involved in the study.

**Table 1:** Common criteria for patient inclusion and exclusion

Inclusion criteria	Exclusion criteria
(1) individuals falling within the age range of 18 to 65 years.	(1) individuals with a present or past occurrence of significant neurological disorders such as Alzheimer’s disease, amyotrophic lateral sclerosis, ischemia, trauma, hepatic encephalopathy, Down’s syndrome, autism, multiple sclerosis, brain neoplasms, Parkinson’s disease, and epilepsy.
(2) the individual fulfills the diagnostic criteria outlined in the DSM-V for depression.	(2) the individual’s psychiatric profile encompasses a range of disorders, such as anxiety, schizophrenia, bipolar disorder, obsessive-compulsive disorder, alcohol and substance abuse, and attention-deficit hyperactivity disorder, either in their current state or throughout their lifetime.
(3) the Hamilton Depression Rating Scale-24 (HAMD-24) yields scores exceeding 20.	(3) individual exhibits a present or cumulative record of enduring infections, inflammatory and immune disorders such as rheumatoid arthritis, inflammatory bowel disease, nephrotic syndrome, systemic lupus erythematosus, multiple sclerosis, autoimmune type I diabetes, asthma, sepsis, pulmonary fibrosis, primary biliary cirrhosis, autoimmune myasthenia gravis, and stroke.
(4) participants reported no utilization of antidepressants, anti-inflammatory agents, or other psychotropic drugs within the preceding three-month period.	(4) the individual is presently undergoing anti-inflammatory therapy.

### Blood sample collection and measurement of serum cytokines

Blood samples were collected from the subjects between 7:00 and 8:00 A.M. and subsequently centrifuged at 1200 × g for 10 min at 4 °C. The resulting supernatant was used for serum samples, which were stored at -80 °C until further analysis. A total of 7 serum cytokines, namely, IL-1β, IL-2, IL-6, IL-8, IL-12, TNF-α, and IFN-γ, were quantified using multiplex bead immunoassays (LXSAHM-10 and LXSAHM-27, R&D Systems for Antibody Detection, Shanghai Universal Biotech Co., Ltd.) following the manufacturer’s instructions. The generation of standard curves involved the utilization of the reference cytokine sample provided in the kit, which subsequently facilitated the calculation of cytokine concentrations in aqueous humor samples. With the exception of a limited number of values falling below the detection limit, all remaining values were found to fall within the interval of the calibration curve.

### Statistical analysis

Statistical analysis was conducted using SPSS (version 17.0; IBM Corp., Armonk, NY, USA). The level of statistical significance was determined at a *P* value < 0.05. The normality of continuous variables was assessed using the Kolmogorov‒Smirnov normality test. For normally distributed data, the independent sample t test or paired sample t test was used, while the Mann‒Whitney U test and Wilcoxon signed-rank test were used for data with a skewed distribution. Quantitative variables that followed a normal distribution are reported as the mean ± standard deviation (SD), while skewed quantitative variables are reported as the median and interquartile range (IQR; 25–75%). The Benjamini‒Hochberg (BH)‒corrected *P* value (FDR-False Discovery Rate) was employed to account for the issue of multiple testing. Receiver operating characteristic (ROC) analysis was performed to estimate the diagnostic performance of serum cytokines in discriminating MDD patients from healthy controls. Pearson’s correlation test was used for correlation analyses. In adherence to convention and to mitigate the undue influence of individual data points, outliers (data points > 3 SD above or below the mean) were excluded from the dataset.

## Results

### Demographic and clinical characteristics of the participants

Table 2 presents a comprehensive overview of the demographic and clinical attributes of both healthy controls and individuals diagnosed with MDD. The current investigation involved the enrollment of 15 female and 8 male participants, in keeping with the established gender disparity in MDD incidence [15, 16]. Twenty-three sex-matched healthy volunteers were included as a control group. No statistically significant differences in age or body mass index (BMI) were observed between the two groups.

**Table 2:** Demographic characteristics of healthy controls and MDD patients

Variables	Healthy controls	MDD patients	t	*P*
Gender (Female/male)	15/8	15/8		
Age	38.91 ± 10.38	39.87 ± 15.80	-0.243	0.810
BMI (kg/m^2^)	23.95 ± 3.06	22.63 ± 3.83	1.286	0.205

### Comparison of serum proinflammatory cytokines between healthy controls and MDD patients

According to the data presented in Table 3, the concentrations of IL-2, IL-6, IL-8, IL-12, TNF-α, and IFN-γ were significantly greater in individuals diagnosed with MDD than in individuals in the control group. Conversely, no statistically significant differences in the levels of IL-1β were detected between the two cohorts.

**Table 3:** Comparison of serum pro-inflammatory cytokines between healthy controls and MDD patients

Variables (pg/ml)	Healthy controls	MDD patients	t/Z	*P*
IL-1β	6.39 ± 0.94	7.55 ± 2.49	-1.999	0.057
IL-2	9.82 ± 1.49	29.08 ± 16.96	-5.306	< 0.001
IL-6	1.52 (1.08, 2.05)	4.31 (2.20, 13.77)	-3.965	< 0.001
IL-8	26.00 ± 21.29	451.24 ± 383.24	-5.197	< 0.001
IL-12	135.30 ± 55.11	222.54 ± 46.79	-5.788	< 0.001
TNF-α	2.88 ± 0.87	4.50 ± 2.43	-2.881	0.008
IFN-γ	10.36 ± 2.39	15.86 ± 4.08	-5.488	< 0.001

### Diagnostic value of different cytokines in discriminating patients with MDD from healthy volunteers

**Fig. 1:**
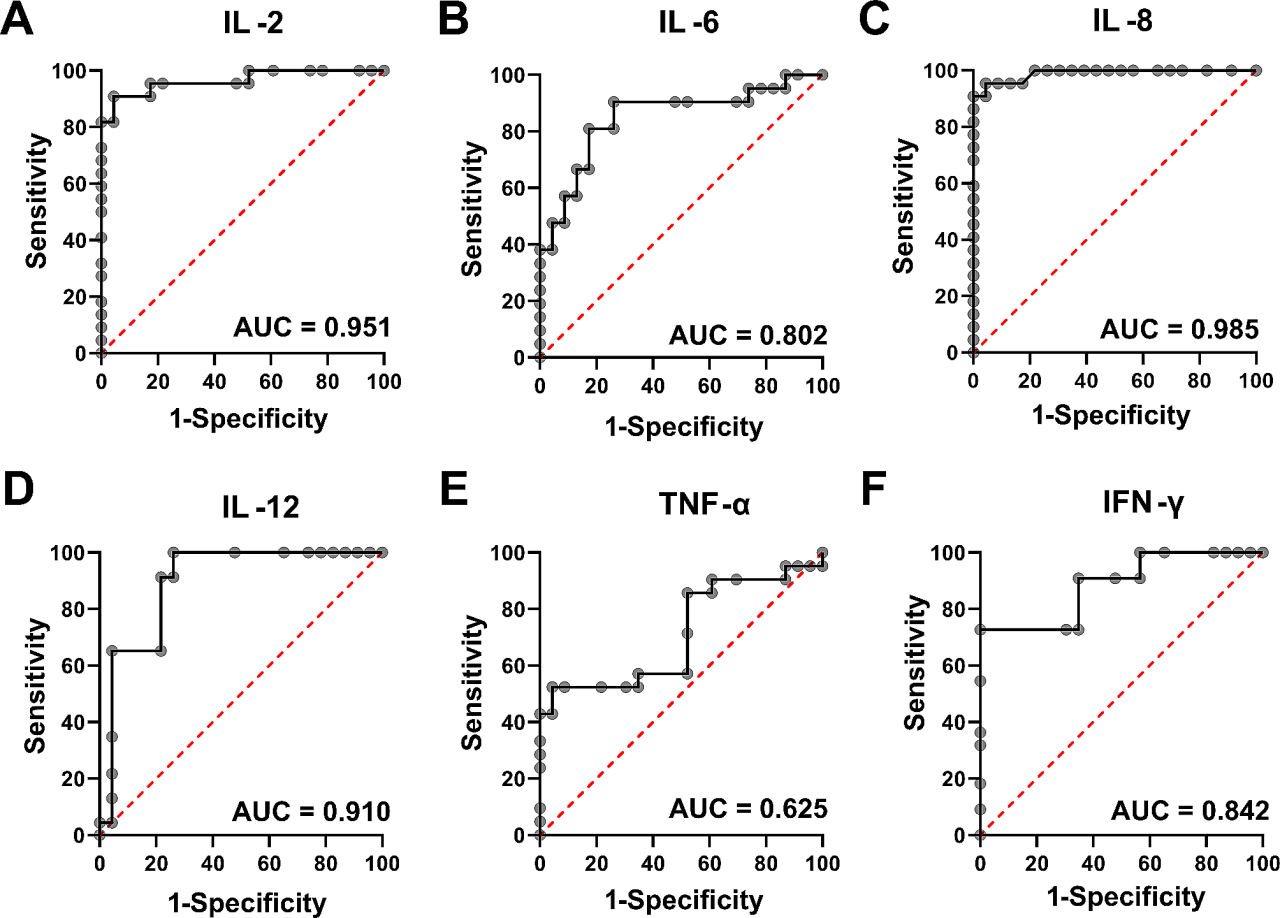
Utilization of ROC curves for the purpose of distinguishing individuals diagnosed with MDD from healthy volunteers. (**A**) ROC curve of IL-2; (**B**) ROC curve of IL-6; (**C**) ROC curve of IL-8; (**D**) ROC curve of IL-12; (**E**) ROC curve of TNF-α; (**F**) ROC curve of IFN-γ.

The diagnostic efficacy of various cytokines in distinguishing patients with MDD from healthy volunteers was assessed through the use of ROC curve analysis (Fig. 1). The findings revealed that 5 cytokines, namely, IL-2, IL-6, IL-8, IL-12, and IFN-γ, exhibited area under the curve (AUC) values exceeding 0.7. Notably, IL-8 demonstrated the most favorable diagnostic performance, as evidenced by the highest AUC value (AUC = 0.985, sensitivity = 93.8%, specificity = 95.7%), followed by IL-2 (AUC = 0.951, sensitivity = 87.5%, specificity = 95.7%) and IL-12 (AUC = 0.910, sensitivity = 93.8%, specificity = 78.3%).

### Relationships between HAMD-24 scores and baseline serum cytokine levels in the MDD group

**Fig. 2:**
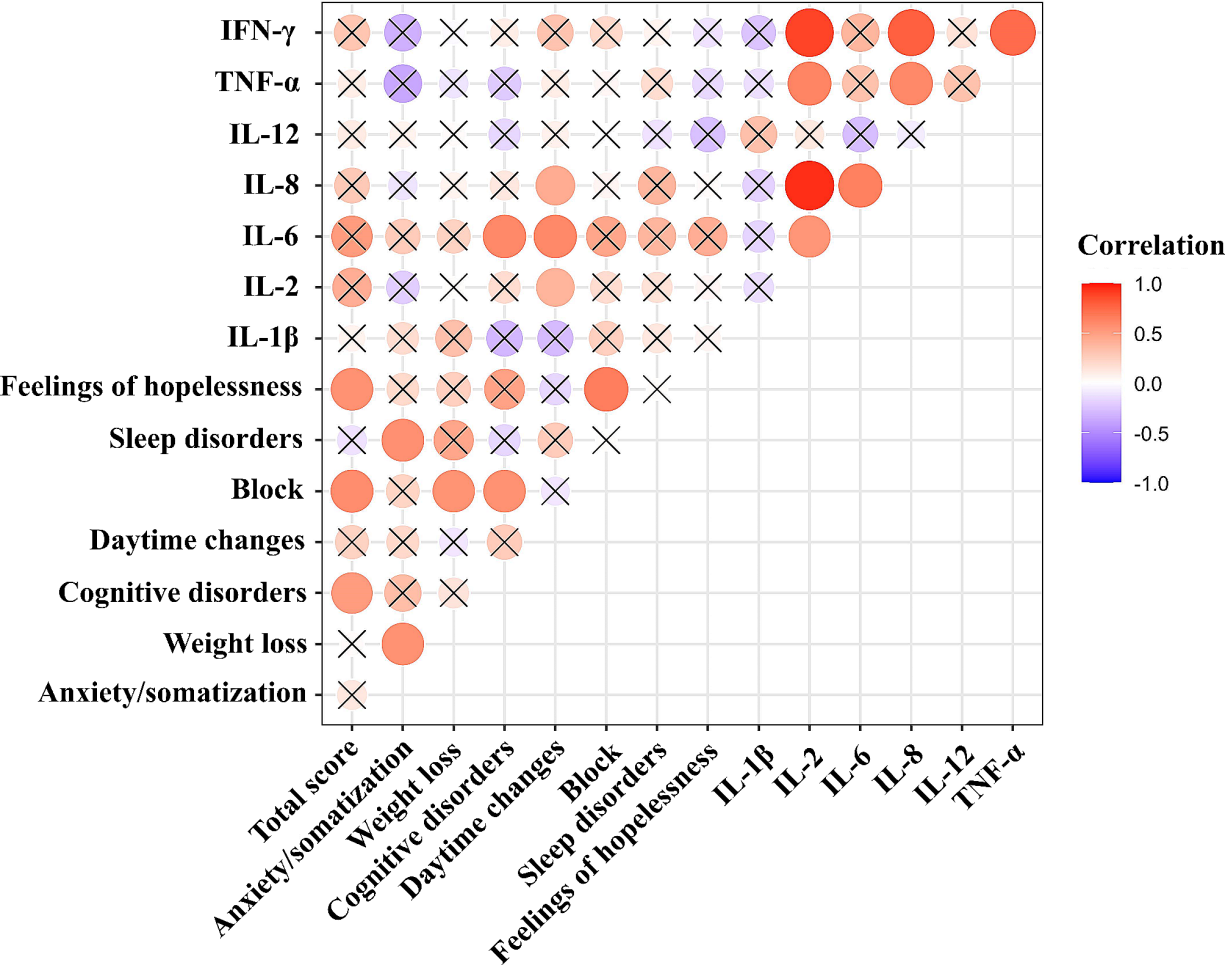
Correlation between HAMD-24 scores and the serum levels of cytokines at baseline in MDD group. ×: no significance

Pearson correlation tests were utilized to analyze the associations between HAMD-24 scores and serum levels of cytokines at baseline in the MDD group (Fig. 2). Daytime changes were positively correlated with IL-2 (*r* = 0.462, *P* = 0.030), IL-6 (*r* = 0.516, *P* = 0.017), and IL-8 (*r* = 0.462, *P* = 0.030) levels, and there was a positive correlation between cognitive disorder scores and IL-6 levels (*r* = 0.492, *P* = 0.023).

### Effect of duloxetine on the HAMD-24 score in patients with MDD

According to the data presented in Table 4, the initial HAMD-24 score was 37.74 ± 11.14. After 4 weeks of duloxetine administration, a significant reduction in the HAMD-24 score was observed, with the score decreasing to 12.57 ± 8.76 (*t* = 13.161, *P* < 0.001).

**Table 4:** Effect of duloxetine on the HAMD-24 scores in patients with MDD

Variables	Before treatment	After treatment	t	*P*
HAMD-24 scores	37.74 ± 11.14	12.57 ± 8.76	13.161	< 0.001

### Effect of duloxetine on the serum levels of proinflammatory cytokines in patients with MDD

After 4 weeks of treatment, the serum levels of IL-8 (*t* = 3.605, *P* = 0.002), IL-12 (*t* = 2.559, *P* = 0.018) and IFN-γ (*t* = 3.567, *P* = 0.002) significantly decreased (Table 5). Conversely, no statistically significant differences were observed in the levels of other cytokines, including IL-1β, IL-2, IL-6, and TNF-α, before and after treatment (*P* > 0.05; Table 5).

**Table 5:** Effect of duloxetine on the serum levels of pro-inflammatory cytokines in patients with MDD

Variables (pg/ml)	Before treatment	After treatment	t/Z	*P*
IL-1β	8.36 ± 3.93	6.96 ± 3.00	1.219	0.237
IL-2	27.43 (15.51, 41.58)	15.51 (13.27, 28.77)	-1.672	0.095
IL-6	12.13 ± 16.58	7.77 ± 9.22	1.212	0.240
IL-8	398.42 (126.14, 786.91)	109.96 (82.99, 250.86)	-2.311	0.021
IL-12	222.89 ± 47.86	183.07 ± 65.95	2.559	0.018
TNF-α	5.33 ± 3.67	5.93 ± 3.58	-0.633	0.534
IFN-γ	16.56 ± 4.95	13.26 ± 4.22	3.567	0.002

## Discussion

The current study aimed to examine the impact of duloxetine treatment on inflammation at the clinical level by investigating the serum levels of 7 proinflammatory cytokines in drug naïve MDD patients before and after 4 weeks of treatment. Three primary findings were observed. First, the serum levels of IL-2, IL-6, IL-8, IL-12, TNF-α, and IFN-γ were significantly greater in MDD patients than in healthy controls. Second, the serum levels of IL-2, IL-6, IL-8, IL-12, and IFN-γ exhibit promising potential as diagnostic biomarkers for distinguishing individuals with MDD from healthy volunteers. Third, the serum levels of IL-8, IL-12, and IFN-γ decreased significantly following duloxetine treatment.

Cytokines are known to exert a significant influence on the onset and progression of psychological disorders, such as depression. Nevertheless, the precise underlying mechanism remains incompletely elucidated and potentially involves the following factors: (1) cytokines modulate intracellular metabolic pathways, thereby promoting oxidative stress and neural apoptosis, ultimately contributing to the manifestation of depression [17]; (2) the integrity of neurotransmitter signaling in the cerebral cortex and hippocampus may be compromised by cytokines, resulting in an imbalance of neurotransmitters and subsequent psychiatric disorders such as depression [18]; and (3) the activation of neuroglia by cytokines has been recognized as a contributing factor to psychiatric disorders [19]. Consequently, investigating the abnormal expression profile of cytokines in the peripheral blood of individuals with MDD could offer a novel approach to understanding the underlying mechanisms of MDD.

The present study revealed an increase in the levels of six proinflammatory cytokines (IL-2, IL-6, IL-8, IL-12, TNF-α, and IFN-γ) in the serum of individuals diagnosed with MDD. Consistent with this, a recent systematic review and meta-analysis encompassing 23 relevant studies (consisting of 1,366 MDD patients and 1,342 controls) revealed a significant increase in peripheral TNF-α levels among depressed individuals compared to controls [20]. Moreover, an updated meta-analysis incorporating data from 82 studies involving a total of 3,212 participants diagnosed with MDD and 2,798 healthy controls revealed that individuals with MDD exhibit elevated peripheral levels of IL-6, IL-10, IL-12, IL-13, and TNF-α compared to their healthy counterparts [21]. Furthermore, additional investigations have shown increased blood levels of IL-1β [22], IL-2 [23], and IL-8 [24] in individuals suffering from depression. These findings contribute valuable data and expand the range of potential serum cytokine-based biomarkers than can be used for an accurate diagnosis of depression.

The HAMD-24 scale includes 24 items, dividing depressive symptoms into 7 categories of factors: anxiety/somatization, weight loss, cognitive disorders, daytime changes, block, sleep disorders, and feelings of hopelessness. In the present study, the relationship between HAMD-24 scores (total score and scores for 7 factors) and the serum levels of cytokines at baseline in the MDD group was investigated. In the present study, the daytime changes in the HAMD-24 score were positively related to the IL-2, IL-6, and IL-8 levels. Moreover, cognitive disorder scores were positively related to IL-6 levels. The precise mechanism underlying the correlation between various core symptoms of depression and distinct proinflammatory factors remains unclear, necessitating additional research to elucidate this relationship.

Further investigation was conducted to explore the potential diagnostic value of these aberrant cytokines in MDD. According to conventional standards, an AUC greater than 0.7 in a ROC analysis is deemed acceptable for clinical application. A comprehensive set of five cytokines, namely, IL-2, IL-6, IL-8, IL-12, and IFN-γ, satisfy the aforementioned condition. Among these factors, IL-2, IL-8, and IL-12 exhibit comparable discriminatory capacity (AUC exceeding 0.9) in distinguishing individuals with MDD from their healthy counterparts. IL-8 demonstrated the most favorable diagnostic performance. IL-8 serves as a crucial inflammatory mediator that is synthesized in various tissues following exposure to inflammatory cytokines. Upon secretion, it attracts lymphocytes and stimulates neutrophil granulocytes, leading to the autocrine production of IL-8 and thereby intensifying the inflammatory response. Compared with other cytokines, IL-8 exhibits a relatively slow degradation rate, allowing it to persist in its active form for an extended period within the immediate cellular environment. This characteristic facilitates its use in research studies in comparison to other interleukins [25]. Recent evidence has indicated a correlation between IL-8 and MDD. In a study involving a cohort of 1037 elderly individuals from the community who were evaluated at baseline and after a 2-year period, it was observed that elevated serum IL-8 levels were linked to depressive symptoms both at the initial assessment and at the follow-up evaluation [26]. Similarly, in another study involving 732 elderly individuals in Korea, depression at the initial assessment was significantly correlated with elevated serum IL-8 levels. Furthermore, the onset of depression during the 2-year follow-up period was significantly linked to an increase in IL-8 levels [27]. In a small-scale meta-analysis involving two studies with a total of 38 patients diagnosed with MDD and 114 healthy controls, it was found that cerebrospinal fluid (CSF) levels of IL-8 were significantly greater in MDD patients than in controls [28]. Shelton et al. conducted a postmortem examination of the brains of individuals with depression, revealing elevated gene expression of IL-8 in the frontal cortex [29]. Collectively, these findings indicate a strong correlation between IL-8 and MDD, indicating its potential utility as a biomarker for the identification of depression.

The anti-inflammatory properties of antidepressant medications have been extensively demonstrated in various studies. Specifically, tricyclic antidepressants such as citalopram, clomipramine, and imipramine have been found to suppress the secretion of the proinflammatory cytokines IL-6, IL-1β, and TNF-α in human monocytes [30]. The effects of fluoxetine, the most commonly prescribed antidepressant in clinical practice, are also attributed to its anti-inflammatory mechanisms. Studies have demonstrated that fluoxetine treatment leads to a significant decrease in the levels of IFN-γ and TNF-α and in the IFN-γ/IL-10 ratio in the blood of healthy individuals [31]. Additionally, several studies have demonstrated the anti-inflammatory effects of ketamine. For instance, one study revealed that ketamine infusion led to a decrease in serum TNF-α levels in patients with treatment-resistant MDD [32]. Another study showed that ketamine was effective in reducing serum IL-6 levels in individuals with MDD [33]. More recently, multiple meta-analyses have been conducted to investigate the impact of antidepressant treatment on peripheral cytokine levels in serum. A meta-analysis comprising 22 studies revealed that treatment with SSRIs resulted in a reduction in proinflammatory peripheral levels of IL-1β and IL-6 [34]. Another meta-analysis indicated that antidepressant treatment decreases peripheral levels of inflammatory cytokines, including IL-4, IL-6, and IL-10, in patients with MDD [35].

To date, there is a lack of research examining the longitudinal impact of duloxetine on different proinflammatory cytokines in individuals diagnosed with MDD. Several animal studies have investigated the effects of duloxetine on various proinflammatory cytokines. For example, it has been reported that the administration of 20 mg/kg duloxetine was able to reduce the increases in TNF-α and IL-1β levels in the hippocampal CA1 region after 28 days of chronic cerebral hypoperfusion [36]. Moreover, duloxetine treatment was found to suppress kainic acid-induced elevations in TNF-α and IL-1β levels in the hippocampus [37]. In addition, pretreatment with duloxetine mitigated the lipopolysaccharide-induced increase in TNF-α in mice [38]. The present study revealed that 4 weeks of duloxetine treatment decreased the levels of IL-8, IL-12, and IFN-γ in MDD patients. However, the precise mechanism through which duloxetine modulates cytokine levels remains uncertain and intricate. The literature has documented the role of cyclic adenosyl monophosphate (cAMP), the hypothalamic‒pituitary‒adrenal (HPA) axis, serotonin metabolism, and neurogenesis in the modulation of cytokine functioning by antidepressants [39, 40]. For example, it has been postulated that cAMP may play a role in mediating the antidepressant effects on cytokine levels in vitro. Studies have demonstrated that the antidepressants imipramine, clomipramine (TCA), and citalopram (SSRI) can elevate intracellular cAMP levels [30]. An increase in the intracellular cAMP concentration in various cell types, including monocytes, macrophages, and microglia, results in a reduction in proinflammatory cytokine production [30, 41, 42]. In terms of duloxetine, peroxisome proliferator-activated receptor gamma (PPARγ), which has anti-inflammatory activity, has been proposed to be involved in the modulation of cytokine level by duloxetine in vivo. It has been reported that IL-6 and TNF-α mRNA expression in the brains of prenatally stressed mice that underwent duloxetine therapy was decreased [43]. Specifically, the study defined the day of sperm positivity as gestational Day 0 (GD0), and pregnant dams assigned to the prenatal stress group were exposed to chronic mild stress from GD4 to GD20. The day of pup birth was designated postnatal Day 0 (PND0), and on PND60, the offspring were administered either duloxetine or vehicle. Injections were administered once daily for 21 days. The results showed that the administration of duloxetine effectively mitigated the increase in the levels of proinflammatory cytokines in the hippocampus, as well as anxiety- and depression-like behaviors, in adult offspring exposed to prenatal stress [43]. Further studies revealed that duloxetine upregulated the protein expression of PPARγ in the prefrontal cortex and hippocampus, indicating its potential to suppress neuroinflammation associated with depression by inhibiting the transcription of proinflammatory genes through PPARγ [44, 45]. The results of the present study suggest that the antidepressant efficacy of duloxetine in individuals with MDD may be attributed, at least in part, to its anti-inflammatory properties, particularly in relation to the peripheral levels of IL-8, IL-12, and IFN-γ, but not IL-1β, IL-2, IL-6, and TNF-α. Since this was a single-center study and the sample size was relatively small, the regulatory effect of duloxetine on the levels of these proinflammatory cytokines should be confirmed by multicentric studies. Given that this was an observational study, additional research is warranted to elucidate the mechanisms through which duloxetine exerts varying regulatory effects on these proinflammatory cytokines.

It is known that a period of at least four weeks of antidepressant treatment is necessary to ascertain the responsiveness of the patient [46]. Consistent with the guidelines for the diagnosis and treatment of depressive disorders in China (the second edition), it is recommended that patients adhere to a minimum of 4 weeks of antidepressant therapy before the effectiveness of the medication is assessed. Additionally, several studies have characterized individuals who respond to antidepressant treatment as those who exhibit at least a 50% reduction in their HAMD total score following 4 weeks of medication therapy [47–49]. Therefore, a 4-week period of antidepressant treatment was selected to assess the impact of duloxetine on serum cytokine levels in the present study. According to the United States Food and Drug Administration (FDA) and the guidelines for the diagnosis and treatment of depressive disorders in China (the second edition), the approved dose range for duloxetine in the treatment of MDD is 40–60 mg/day [50–52]. Thus, MDD patients who received duloxetine treatment at a dosage range of 40–60 mg/day were selected for the present study.

There are several noteworthy limitations in the current study that warrant attention. First, it is important to acknowledge the relatively small sample size of this study, which was conducted at a single center. Second, the measurement of serum cytokines was limited to before and after the 4-week treatment period, thus failing to capture any dynamic changes in cytokine levels during treatment or the potential effects of long-term (8–12 weeks) antidepressant treatment on cytokine levels. Third, the assessment of serum concentrations of duloxetine was not conducted, thereby precluding the ability to examine any correlation between duloxetine concentrations and cytokine levels. Fourth, due to a lack of published data and preliminary data, we were not able to form a meaningful sample size calculation.

In summary, our findings indicate that several proinflammatory cytokines, namely, IL-2, IL-6, IL-8, IL-12, and IFN-γ, have potential as diagnostic biomarkers for distinguishing patients with MD from healthy individuals. Additionally, our results demonstrate that a 4-week treatment with duloxetine can effectively reduce the elevated levels of IL-8, IL-12, and IFN-γ in MDD patients. These findings provide further evidence supporting the hypothesis that inflammation may play a role in the development of depression and suggest that the anti-inflammatory properties of antidepressants may contribute to their therapeutic effects in MDD patients.

## Data Availability

The datasets generated during and/or analyzed during the current study are available from the corresponding author on reasonable request.

## References

[1] McCarron R, Shapiro B, Rawles J, Luo J (2021). Depression. Ann Intern Med.

[2] Huang Y, Wang Y, Wang H, Liu Z, Yu X, Yan J, Yu Y, Kou C, Xu X, Lu J (2019). Prevalence of mental disorders in China: a cross-sectional epidemiological study. Lancet Psychiatry.

[3] Beurel E, Toups M, Nemeroff C (2020). The bidirectional relationship of depression and inflammation: double trouble. Neuron.

[4] Goldsmith D, Bekhbat M, Mehta N, Felger J (2023). Inflammation-related functional and Structural Dysconnectivity as a pathway to psychopathology. Biol Psychiatry.

[5] Osimo E, Baxter L, Lewis G, Jones P, Khandaker G (2019). Prevalence of low-grade inflammation in depression: a systematic review and meta-analysis of CRP levels. Psychol Med.

[6] Dowlati Y, Herrmann N, Swardfager W, Liu H, Sham L, Reim E, Lanctôt K (2010). A meta-analysis of cytokines in major depression. Biol Psychiatry.

[7] Talarowska M, Szemraj J, Gałecki P (2016). The role of interleukin genes in the course of depression. Open Med (Warsaw Poland).

[8] Esalatmanesh S, Kashani L, Khooshideh M, Moghaddam H, Ansari S, Akhondzadeh S (2023). Efficacy and safety of celecoxib for treatment of mild to moderate postpartum depression: a randomized, double-blind, placebo-controlled trial. Arch Gynecol Obstet.

[9] Horikawa H, Kato T, Mizoguchi Y, Monji A, Seki Y, Ohkuri T, Gotoh L, Yonaha M, Ueda T, Hashioka S (2010). Inhibitory effects of SSRIs on IFN-γ induced microglial activation through the regulation of intracellular calcium. Prog Neuro-psychopharmacol Biol Psychiatry.

[10] Kubera M, Lin A, Kenis G, Bosmans E, van Bockstaele D, Maes M (2001). Anti-inflammatory effects of antidepressants through suppression of the interferon-gamma/interleukin-10 production ratio. J Clin Psychopharmacol.

[11] Ohgi Y, Futamura T, Kikuchi T, Hashimoto K (2013). Effects of antidepressants on alternations in serum cytokines and depressive-like behavior in mice after lipopolysaccharide administration. Pharmacol Biochem Behav.

[12] Yao L, Pan L, Qian M, Sun W, Gu C, Chen L, Tang X, Hu Y, Xu L, Wei Y (2020). Tumor necrosis Factor-α variations in patients with major depressive disorder before and after antidepressant treatment. Front Psychiatry.

[13] Ankarfeldt M, Petersen J, Andersen J, Li H, Motsko S, Fast T, Hede S, Jimenez-Solem E (2021). Exposure to duloxetine during pregnancy and risk of congenital malformations and stillbirth: a nationwide cohort study in Denmark and Sweden. PLoS Med.

[14] Zheng Y, Zhao J, Phillips M, Liu J, Cai M, Sun S, Huang M (1988). Validity and reliability of the Chinese Hamilton Depression Rating Scale. Br J Psychiatry: J Mental Sci.

[15] Kessler R, Berglund P, Demler O, Jin R, Koretz D, Merikangas K, Rush A, Walters E, Wang P (2003). The epidemiology of major depressive disorder: results from the National Comorbidity Survey replication (NCS-R). JAMA.

[16] Kessler R, Berglund P, Demler O, Jin R, Merikangas K, Walters E (2005). Lifetime prevalence and age-of-onset distributions of DSM-IV disorders in the National Comorbidity Survey Replication. Arch Gen Psychiatry.

[17] Jones N, Blagih J, Zani F, Rees A, Hill D, Jenkins B, Bull C, Moreira D, Bantan A, Cronin J (2021). Fructose reprogrammes glutamine-dependent oxidative metabolism to support LPS-induced inflammation. Nat Commun.

[18] Duman R, Sanacora G, Krystal J (2019). Altered connectivity in Depression: GABA and glutamate neurotransmitter deficits and reversal by Novel treatments. Neuron.

[19] Najjar S, Pearlman D, Alper K, Najjar A, Devinsky O (2013). Neuroinflammation and psychiatric illness. J Neuroinflamm.

[20] Islam M, Sohan M, Daria S, Masud A, Ahmed M, Roy A, Shahriar M (2023). Evaluation of inflammatory cytokines in drug-naïve major depressive disorder: a systematic review and meta-analysis. Int J ImmunoPathol Pharmacol.

[21] Köhler C, Freitas T, Maes M, de Andrade N, Liu C, Fernandes B, Stubbs B, Solmi M, Veronese N, Herrmann N (2017). Peripheral cytokine and chemokine alterations in depression: a meta-analysis of 82 studies. Acta Psychiatrica Scandinavica.

[22] Mota R, Gazal M, Acosta B, de Leon P, Jansen K, Pinheiro R, Souza L, Silva R, Oses J, Quevedo L (2013). Interleukin-1β is associated with depressive episode in major depression but not in bipolar disorder. J Psychiatr Res.

[23] Jeenger J, Singroha V, Sharma M, Mathur D (2018). C-reactive protein, brain-derived neurotrophic factor, interleukin-2, and stressful life events in drug-naive first-episode and recurrent depression: a cross-sectional study. Indian J Psychiatry.

[24] Suarez E, Krishnan R, Lewis J (2003). The relation of severity of depressive symptoms to monocyte-associated proinflammatory cytokines and chemokines in apparently healthy men. Psychosom Med.

[25] Tsai S (2020). Role of interleukin 8 in depression and other psychiatric disorders. Prog Neuropsychopharmacol Biol Psychiatry.

[26] Baune B, Smith E, Reppermund S, Air T, Samaras K, Lux O, Brodaty H, Sachdev P, Trollor J (2012). Inflammatory biomarkers predict depressive, but not anxiety symptoms during aging: the prospective Sydney Memory and Aging Study. Psychoneuroendocrinology.

[27] Kim J, Stewart R, Kim J, Kang H, Bae K, Kim S, Shin I, Yoon J (2018). Changes in pro-inflammatory cytokine levels and late-life depression: a two year population based longitudinal study. Psychoneuroendocrinology.

[28] Wang A, Miller B (2017). Meta-analysis of Cerebrospinal Fluid Cytokine and Tryptophan Catabolite alterations in Psychiatric patients: comparisons between Schizophrenia, bipolar disorder, and Depression. Schizophr Bull.

[29] Shelton R, Claiborne J, Sidoryk-Wegrzynowicz M, Reddy R, Aschner M, Lewis D, Mirnics K (2010). Altered expression of genes involved in inflammation and apoptosis in frontal cortex in major depression. Mol Psychiatry.

[30] Xia Z, DePierre J, Nässberger L (1996). Tricyclic antidepressants inhibit IL-6, IL-1 beta and TNF-alpha release in human blood monocytes and IL-2 and interferon-gamma in T cells. Immunopharmacology.

[31] Maes M, Kenis G, Kubera M, Baets M, Steinbusch H, Bosmans E (2005). The negative immunoregulatory effects of fluoxetine in relation to the cAMP-dependent PKA pathway. Int Immunopharmacol.

[32] Chen M, Li C, Lin W, Hong C, Tu P, Bai Y, Cheng C, Su T (2018). Rapid inflammation modulation and antidepressant efficacy of a low-dose ketamine infusion in treatment-resistant depression: a randomized, double-blind control study. Psychiatry Res.

[33] Kiraly D, Horn S, Van Dam N, Costi S, Schwartz J, Kim-Schulze S, Patel M, Hodes G, Russo S, Merad M (2017). Altered peripheral immune profiles in treatment-resistant depression: response to ketamine and prediction of treatment outcome. Transl Psychiatry.

[34] Wang L, Wang R, Liu L, Qiao D, Baldwin D, Hou R (2019). Effects of SSRIs on peripheral inflammatory markers in patients with major depressive disorder: a systematic review and meta-analysis. Brain Behav Immun.

[35] Więdłocha M, Marcinowicz P, Krupa R, Janoska-Jaździk M, Janus M, Dębowska W, Mosiołek A, Waszkiewicz N, Szulc A (2018). Effect of antidepressant treatment on peripheral inflammation markers - a meta-analysis. Prog Neuro-psychopharmacol Biol Psychiatry.

[36] Park J, Lee C (2017). Neuroprotective effect of Duloxetine on Chronic Cerebral Hypoperfusion-Induced hippocampal neuronal damage. Biomol Ther (Seoul).

[37] Choi H, Park J, Ahn J, Hong S, Cho J, Won M, Lee C (2015). The anti-inflammatory activity of duloxetine, a serotonin/norepinephrine reuptake inhibitor, prevents kainic acid-induced hippocampal neuronal death in mice. J Neurol Sci.

[38] Ohgi Y, Futamura T, Kikuchi T, Hashimoto K (2012). Effects of antidepressants on alternations in serum cytokines and depressive-like behavior in mice after lipopolysaccharide administration. Pharmacol Biochem Behav.

[39] Kopschina Feltes P, Doorduin J, Klein H, Juárez-Orozco L, Dierckx R, Moriguchi-Jeckel C, de Vries E (2017). Anti-inflammatory treatment for major depressive disorder: implications for patients with an elevated immune profile and non-responders to standard antidepressant therapy. J Psychopharmacol (Oxford England).

[40] Vojvodic J, Mihajlovic G, Vojvodic P, Radomirovic D, Vojvodic A, Vlaskovic-Jovicevic T, Peric-Hajzler Z, Matovic D, Dimitrijevic S, Sijan G (2019). The impact of immunological factors on Depression Treatment - Relation between antidepressants and Immunomodulation agents. Open Access Macedonian J Med Sci.

[41] Hashioka S, Klegeris A, Monji A, Kato T, Sawada M, McGeer P, Kanba S (2007). Antidepressants inhibit interferon-gamma-induced microglial production of IL-6 and nitric oxide. Exp Neurol.

[42] Maes M (2001). The immunoregulatory effects of antidepressants. Human Psychopharmacol.

[43] Zhang X, Wang Q, Wang Y, Hu J, Jiang H, Cheng W, Ma Y, Liu M, Sun A, Zhang X (2016). Duloxetine prevents the effects of prenatal stress on depressive-like and anxiety-like behavior and hippocampal expression of pro-inflammatory cytokines in adult male offspring rats. Int J Dev Neuroscience: Official J Int Soc Dev Neurosci.

[44] Kapadia R, Yi J, Vemuganti R (2007). Mechanisms of anti-inflammatory and neuroprotective actions of PPAR-gamma agonists. Front Biosci.

[45] Martín-Hernández D, Bris Á, MacDowell K, García-Bueno B, Madrigal J, Leza J, Caso J (2016). Modulation of the antioxidant nuclear factor (erythroid 2-derived)-like 2 pathway by antidepressants in rats. Neuropharmacology.

[46] Macher J, Crocq M (2004). Treatment goals: response and nonresponse. Dialog Clin Neurosci.

[47] Hsieh M, Lin C, Lee C, Huang T. Abnormal brain-derived neurotrophic factor exon IX promoter methylation, protein, and mRNA levels in patients with major depressive disorder. J Clin Med 2019, 8(5). 10.3390/jcm8050568 PMC6571872 31027379

[48] Wang Q, Tian S, Zhao P, Cao Q, Lu Q, Yao Z (2022). Association between antidepressant efficacy and interactions of three Core Depression-related brain networks in major depressive disorder. Front Psychiatry.

[49] Schüle C, Baghai T, Eser D, Häfner S, Born C, Herrmann S, Rupprecht R (2009). The combined dexamethasone/CRH test (DEX/CRH test) and prediction of acute treatment response in major depression. PLoS ONE.

[50] Westanmo A, Gayken J, Haight R (2005). Duloxetine: a balanced and selective norepinephrine- and serotonin-reuptake inhibitor. Am J Health Syst Pharm.

[51] Khan A, Macaluso M (2009). Duloxetine for the treatment of generalized anxiety disorder: a review. Neuropsychiatr Dis Treat.

[52] Mallinckrodt C, Prakash A, Andorn A, Watkin J, Wohlreich M (2005). Duloxetine for the treatment of major depressive disorder: a closer look at efficacy and safety data across the approved dose range. J Psychiatr Res.

